# Escherichia coli Cellular Activity and Frontal Trizonal Evaluation of Microspace Between Implants and Abutments Under Calibrated Cyclic Stress

**DOI:** 10.7759/cureus.44816

**Published:** 2023-09-07

**Authors:** Anantha Nayagi Thivya Rajadurai, Hariharan Ramakrishnan, Jayakrishnakumar Sampathkumar, Vallabh Mahadevan, Shivakumar Baskaran, Vidhya Jeyapalan, Maniamuthu Ragupathi

**Affiliations:** 1 Department of Prosthodontics and Implantology, Ragas Dental College and Hospital, Chennai, IND; 2 Department of Periodontics and Implantology, Ragas Dental College and Hospital, Chennai, IND

**Keywords:** microbial analysis, oral microbiology, dental titanium implant, titanium, e coli: escherichia coli, zirconia, dental implant-abutment design, implant abutment interface, ceramic-reinforced peek

## Abstract

Aim: To evaluate microspace and microleakage between implant and abutments subjected to pre- and post-calibrated cyclic stress.

Materials and methods: Twelve screw-retained implant prostheses with BioHPP polyetheretherketone (PEEK) abutment (Noris Dental Implant System Ltd., Nesher, Israel) (Group I) and 12 screw-retained implant prostheses with computer-assisted design/computer-assisted manufacturing (CAD/CAM) milled zirconia abutment (DentGallop, Houston, TX, USA) (Group II) were connected to their respective implant, and the prosthetic screw was torqued to 30N/cm (Noris). The microspace was evaluated using scanning electron microscopy (SEM; TeScan, Brno, Czech Republic). Twenty-four samples were then induced to cyclic stress (Lokesh Industries, Pune, India) simulating 180 days duration of oral stress. The microspaces (Group IA and Group IIB) were measured post-cyclic stress. Group I and II were again renamed into Group Ia and Group IIb for microbial study. Both implant assemblies were immersed in fresh soybean casein digest broth (SCDB) (Himedia, Mumbai, India) and subsequently inoculated with 1.0µL E. coli suspension (Himedia) at the open end and incubated at 37ºC for seven days. After the incubation period, cellular activity was determined by the spread plate method, and total colony-forming units (CFU) were calculated. The results were evaluated using independent T and Mann-Whitney tests.

Result: Average and microspace at the implant-abutment junction of Group I samples in the front right was 12.98µm, center 13.76µm, front left 13.22µm, and in Group II samples, the front right was 18.52µm, center 17.84µm, front left 18.58µm.After being subjected to cyclic loading, the mean levels of the vertical microgap for Group IA samples were: in the front right region 10.37µm, in the center 9.34µm, in the front left 10.51µm and in Group IIB samples front right was 14.59µm, center 13.39µm, front left 13.8µm. Independent t-tests showed insignificant differences between the two groups. The median value of microbial leakage of Group Ia samples after cyclic loading was 30 x 10^3^ CFU/ml, and Group IIb samples were 42 x 10^3^ CFU/ml and were significant.

Conclusion: There was minimal variation in the mean microspace between the BioHPP PEEK abutment and CAD/CAM milled zirconia abutment, and it was insignificant before and after cyclic stress. BioHPP PEEK abutment-titanium implant interfaces showed significantly decreased microbial leakage than CAD/CAM milled zirconia abutment-titanium implant interfaces after cyclic stress.

## Introduction

Implants provide enduring replacement of the missing teeth. In recent days, the primary purpose of implant dentistry has been to provide prosthetic restoration with adequate functional and esthetic results [1].

Titanium is the material of choice for dental implants, attributable to various unique mechanical and biological characteristics. Titanium implant fracture is rare, as it is highly resistant to external forces. Though titanium alloys are similar in strength to steel, they are also around 45% lighter, making them ideal for dental implants. The unique property of titanium implants is that they have rare allergic reactions and are generally considered non-toxic. The success rate of implants lies in the property of osseointegration. Only a few materials bring about osseointegration, titanium being one of them. Titanium implants become tightly integrated into the bone, making it solid and stable [2]. Titanium implants also exhibit astonishing advantages due to their oral biologic acceptance, anti-corrosive nature, and low Young’s modulus [3].

Zirconia abutment over titanium implants offers an esthetic alternative to metal implant abutments. Zirconia abutments have the tremendous advantage of improving their mechanical strength and reliability. Zirconia abutments have good mechanical properties primarily due to stress-related phase changes. The other unique properties of zirconia abutments include excellent biocompatibility, oral anticorrosion expression, and highly functional load bearing. This enabled the enhanced use of Zirconia as a biomaterial in dentistry. They also promote good gingival health around implants by reducing the inflammatory response [4]. Zirconia has a higher healing potential for soft tissues around the implant. Zirconia exhibited clinically better hard and soft tissue integration. The mucosa around the zirconia abutment presented stable conditions after five months of healing [5].

BioHPP (Noris Dental Implant System Ltd., Nesher, Israel) polyetheretherketone (PEEK) is a thermoplastic, luminous material that can withstand high melting temperatures. Literature evidence reveals that the physical property, such as the elastic modulus of PEEK, is 3.6GPa. It can be further upgraded by incorporating carbon fibers, enhancing the elastic modulus to 18GPa.This could mimic the property of cortical bone (15GPa) [6]. PEEK materials reduce the abutment stresses, minimize occlusal forces on tooth structures, and harmonize the surrounding hard and soft tissues. Comparatively, PEEK exhibits fewer hypersensitive and allergic reactions. Periimplantitis is comparatively lesser due to the influence of biofilm structure [7].

Though dental implants have a high success rate, it has certain limitations, peri-implant pathology exists, and the microgap can be an etiological factor [8]. Microspace is the space existing between the implant fixture and the abutment. It can be a source of future microbial complications. Biological problems include peri-implant mucosal inflammation, periimplantitis, and crestal bone reduction. Mechanical problems include abutment screw loosening, abutment fracture, and fixture loss [9]. Microgap between the implant-abutment interface is estimated using different techniques: probing by dental explorer, periotest device, direct X-ray observation, scanning electron microscope (SEM), optical microscope (OM), 3D microtomography technology, and optics coherence tomography. Scanning electron microscopy can also perform selective analysis of zones on the given sample [10].

Bacterial microleakage and colonization occur at the interface [11]. A microspace at the implant-abutment zone can invite microbial flora to multiply at the junction, resulting in crestal bone loss [12]. Peri-implantitis is mainly caused by bacterial infiltration around the implant abutment (IA) junction. It is caused by gram-negative bacteria, which also induce periodontitis [13].

Escherichia coli (E. coli) is a facultative anaerobic gram-negative, coliform bacterium. It is associated with early implant failure. E. coli is an expedient pathogen and resides below crestal gingiva in patients with periodontitis [14].

An early implant failure is mainly associated with the inability to achieve osseointegration. Various microbial species are associated with implant failure, namely Porphyromonas gingivalis, Fusobacterium species, and Candida organisms. Many methods have been employed for analyzing microleakage, such as turbidity of broth, DNA checkerboard, actual time-based reverse transcription polymerase enzyme reaction, staining methods, and colony studies [15]. Currently, limited literature is available to link implant failure and E. coli as causative organisms. Periimplantitis is caused by bacterial growth around the IA junction [13].

In this study, soybean casein digest broth (SCDB) was used as a medium for inoculation. The haziness of the broth evaluated the bacterial penetration around the tissues, and total colony-forming units (CFU) were counted [16].

The proposed hypothetical statement was there should not be differences in vertical microspace and microbioleakage between pre-machined BioHPP PEEK abutment and computer-assisted design/computer-assisted manufacturing (CAD/CAM) milled zirconia abutment after cyclic stress.

## Materials and methods

A 20mm x 30mm x 20mm cube of stainless steel is made by milling the large metal block (Neema Industries, Mumbai, India); acrylic blocks were fabricated and used in this study.

Polymethyl hydrogen siloxane (base) and divinyl polysiloxane (catalyst), with two different consistencies of addition silicone (Aquasil; Dentsply, Charlotte, NC, USA), are used for taking the index of stainless steel die. Take equal amounts of catalyst and base using color-coded measuring spoons and knead with fingertips until the color of the mix is uniform. The mixed condensed polysiloxane and light body addition silicone were placed over the stainless steel die. A metal holder holds the polysiloxane material and is allowed to stand until set. Before removal, ensure the impression is firm, resilient, and non-tacky. After setting, it was detached from the index and examined for defects. The putty index thus obtained was used to prepare specimens of standardized dimensions for this study.

The dental surveyor (Bego, Bremen, Germany) positioned the silicone mold to parallel the base to the ground. Using a spirit level indicator (Jinhua HengDa Tools, Zhejiang, China), the survey platform was positioned parallel to the floor. Using a hand hex driver, a cover screw (Bioline Dental Implant System Ltd., Migdal Tefen, Israel) was placed on the implant. Using a straight mandrel, an implant with a cover screw was affixed to the surveyor's arm's vertical axis. The implant abutment platform was positioned 3mm above the silicone mold surface, and the surveying arm was adjusted to place the implant in the silicone mold's center. After placing the implant, clear auto-polymerizing acrylic resin (D.P.I., Bangalore, India) was used to fill the surrounding space. The implant abutment platform was 3mm above the resin block's surface when filling the silicone mold. The resin block was removed from the silicone mold after the resin had time to polymerize. Twenty-four clear acrylic blocks were made, each stabilizing one implant.

**Figure 1:**
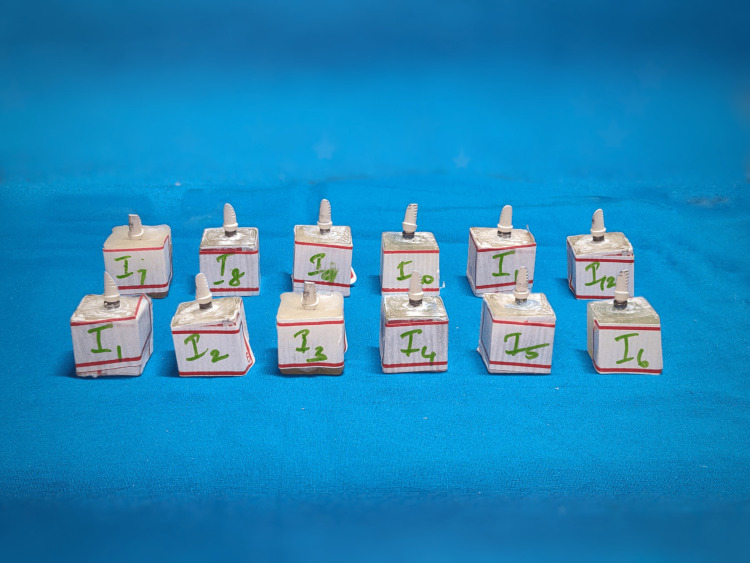
Group I implants with abutments

**Figure 2:**
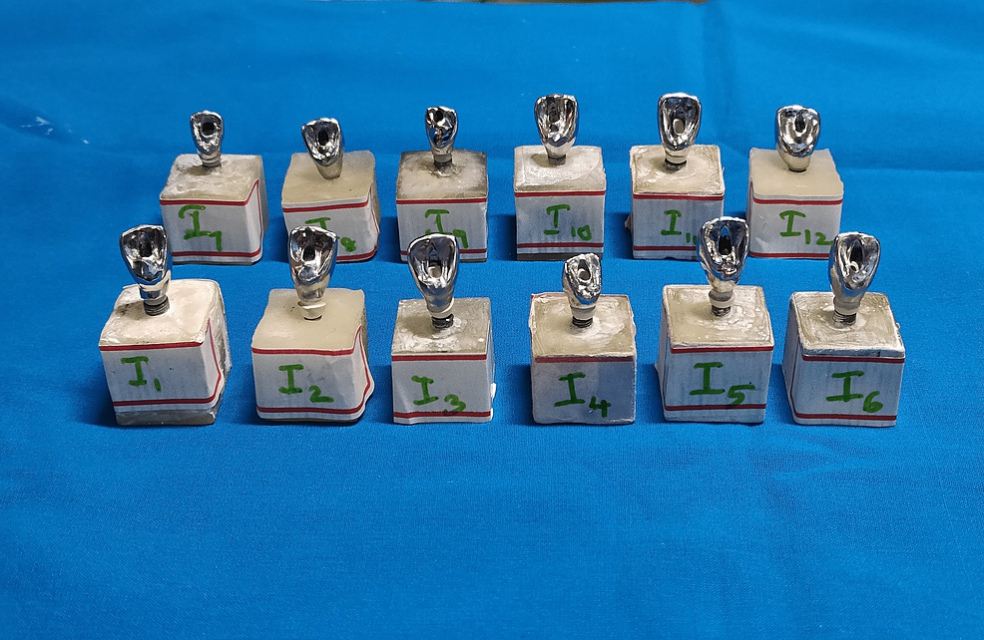
Group I completed samples with crowns

Twelve internal hexagon BioHPP PEEK abutments and 12 CAD/CAM milled zirconia abutments (DentGallop, Houston, TX, USA) were used in the current investigation. Based on the abutments used, the samples placed and enumerated were designated to two test groups. Group I test samples are composed of BioHPP PEEK abutments that were connected to their respective implants first and then loaded with screw Ni-Cr crowns (Figures 1, 2). Group II test samples composed of CAD/CAM milled zirconia abutments were connected to the implants.

**Figure 3:**
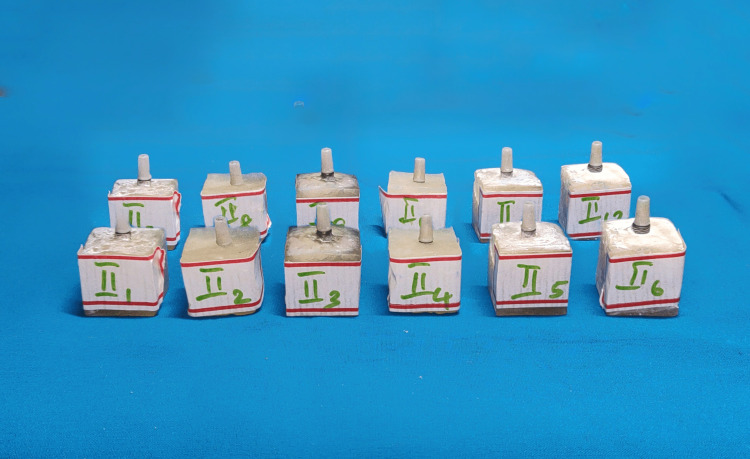
Group II implants with abutments

**Figure 4:**
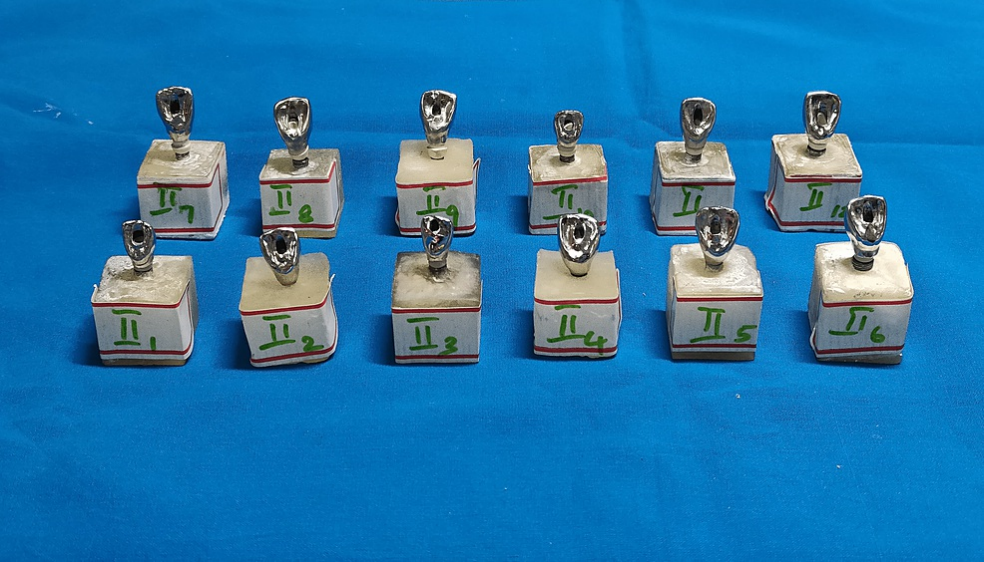
Group II completed samples with crowns

Twenty-four screw-retained cast nickel-chromium (Ni-Cr) crowns were obtained for each implant analog assembly using palatal screw access. Each Ni-Cr crown was chosen and fixed to its respective implant by manually torquing the prosthetic screw to 30N/cm. Crowns were thoroughly inspected for seating and marginal accuracy (Figures 3, 4).

**Figure 5:**
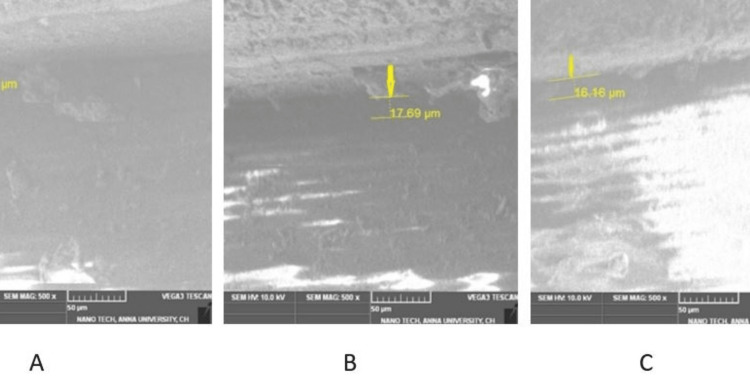
Frontal trizonal images of Group I (A- front right, B- front left, C- center)

**Figure 6:**
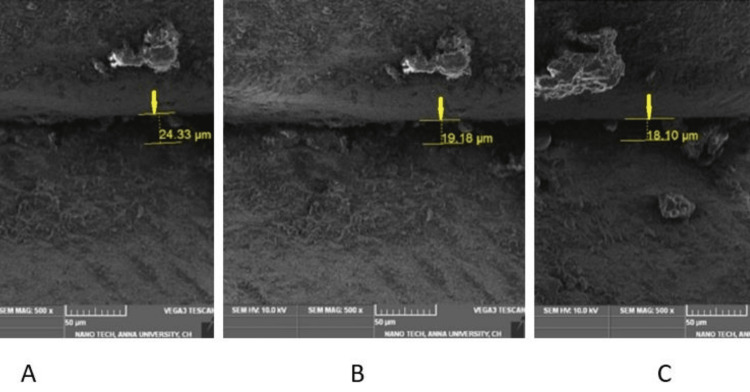
Frontal trizonal images of Group II (A- front right, B- front left, C- center)

The test samples were placed in an SEM source (TeScan, Brno, Czech Republic); the vertical microspace was measured at three different zones: left corners, center, and right corners using digital images (SEM TeScan's Essence software). In this study, all test samples were evaluated under 500x magnification. SEM images were transferred to this file. The vertical microgaps were measured for Group I and Group II test samples (Figures 5, 6).

**Figure 7:**
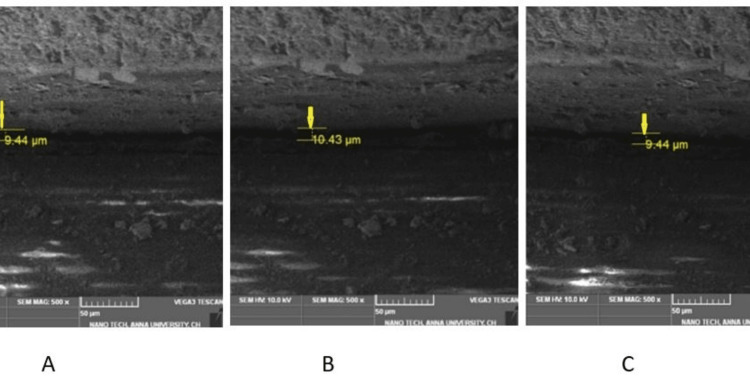
Frontal trizonal images of Group IA (A- front right, B- front left, C- center)

**Figure 8:**
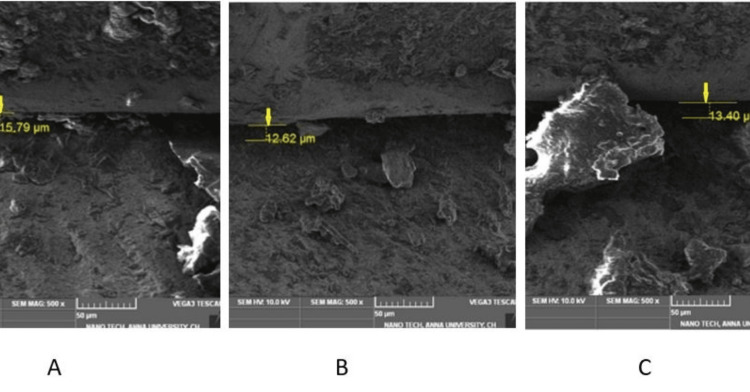
Frontal trizonal images of Group IIB (A- front right, B- front left, C- center)

Calibrated cyclic stress delivery was performed for all test samples (Group I and Group II) using a cyclic stress delivery machine (Lokesh Industries, Chennai, India) simulating oral functions. Test samples with the prosthesis were positioned in the platform and secured at an angle of 30º for the anterior crown. The needle was oriented to contact the cingulum portion of the incisor crown. A cyclic stress delivery of 00 N was applied to each sample for 150,000 cycles, simulating six months of function. The cycle was continued for 24 hours with the discontinuance of one hour every 12 hours. After cyclic loading, existing groups were designated Group IA and Group IIB (Figures 7, 8).

After being subjected to cyclic stress delivery, the samples (Group IA and Group IIB) were re-evaluated at three different points to measure vertical microspace.

Group I and II were further subdivided into Group Ia and Group IIb for microbial study. The test samples (Group a and Group b) were sterilized by autoclaving at 121ºC for 15 minutes under 15 lbs pressure. Implant assemblies were immersed in SCDB (Himedia, Mumbai, India). The bacterial test strain ATCC 25922 Escherichia coli (Himedia) was revived from glycerol at -20ºC by subculturing onto blood agar medium (bioMérieux, Craponne, France) incubated at 37ºC for 24 hours. The initial inoculum was prepared by suspending identical colonies of test strain in 3ml SCDB. The inoculum turbidity was adjusted to OD600nm0.5M Farland standard -108 CFU/ml using DensiCHEKTM (bioMérieux). Both groups (Group a and Group b) were immersed in fresh SCDB, inoculated with 1.0µl E. coli suspension at the open end, and incubated at 37ºC for seven days. During the incubation period, the turbidity of the inoculums was observed in subsequent days.

**Figure 9:**
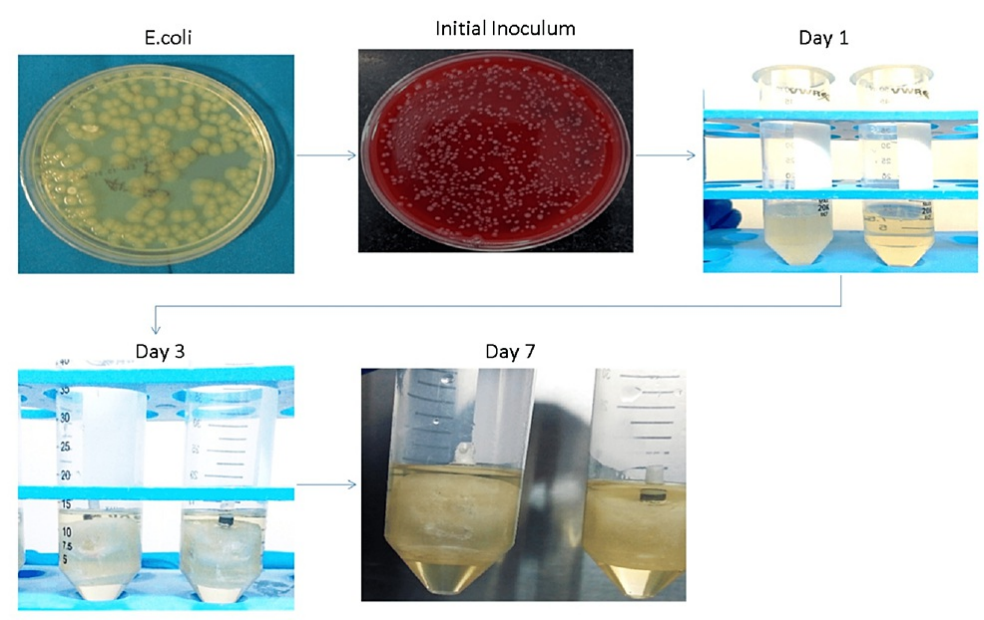
Sequence of E. coli incubation

After the incubation period, microleakage was determined by the spread plate method, and 100µl test SCDB broth was applied and cultured using an L rod. The total number of colonies formed was counted using a digital counter (Figure 9).

The total colony forming unit (TCFU), per ml was calculated as total number of colonies counted divided by dilution factor.

The results were analyzed using the SPSS package v27 (IBM Corp., Armonk, NY, USA). An independent t-test was used to analyze the microspace between the two groups before cyclic loading and the same groups after cyclic loading. Mann Whitney U Test was used to analyze microbioleakage between Group Ia and Group IIb.

## Results

The mean microspace value at the IA junction of Group I samples before cyclic stress was 13.22µm. Group II samples were found to be 18.52µm and were insignificant. The mean microspace value at the IA junction of Group IA samples after cyclic stress was 10.37µm, and Group IIB samples were 13.8µm and were insignificant (Table 1).

**Table 1:** represents significant difference in mean vertical microspace values for front right, center, and front left. *P< 0.05 is considered significant

Microspace	Group I		P value	Group II		P value	Group 1A			Group IIB		
	Mean (µm)	SD (µm)		Mean (µm)	SD (µm)		Mean (µm)	SD (µm)	P Value	Mean (µm)	SD (µm)	P Value
Front right	12.98	2.31	0.7	18.52	1.58	0.68447	10.37	1.14	0.69166	14.59	1.51	0.67247
Center	13.76	2.26	0.58	17.84	1.58	0.68708	9.34	1.11	0.71605	13.39	1.58	0.71335
Front left	13.22	1.96	0.605	18.58	1.62	0.69377	10.51	0.98	0.68139	13.8	1.74	0.70175
Average	13.22	0.18896	0.5	18.52	0.02	0.5	10.37	0.08	0.5	13.8	0.12	0.8

For E. coli cellular activity on the seventh day of incubation, post cyclic stress, the median value of microbioleakage of Group Ia samples was 30 x 103CFU/ml. The median value of microbioleakage of Group IIb samples after cyclic loading was 42 x 103CFU/ml. The median values of Group Ia samples were less than those of Group IIb samples and were significant (Tables 2, 3, 4).

**Table 2:** Microbial leakage of Group Ia samples for E. coli

Samples	Number of colonies (CFU/ml)
1	34 x 10^3^
2	38 x 10^3^
3	26 x 10^3^
4	32 x 10^3^
5	34 x 10^3^
6	36 x 10^3^
7	28 x 10^3^
8	29 x 10^3^
9	30 x 10^3^
10	30 x 10^3^
11	27 x 10^3^
12	22 x 10^3^

**Table 3:** Microbial leakage of Group IIb samples for E. coli

Samples	Number of colonies (CFU/ml)
1	45 x 10^3^
2	39 x 10^3^
3	42 x 10^3^
4	52 x 10^3^
5	51 x 10^3^
6	40 x 10^3^
7	42 x 10^3^
8	42 x 10^3^
9	52 x 10^3^
10	38 x 10^3^
11	40 x 10^3^
12	46 x 10^3^

**Table 4:** Mann Whitney U Test used for comparison of microbial leakage for Group Ia and Group IIb after cyclic loading *P< 0.05 is considered significant

Groups	Median	P Value
Group Ia	30 x 10^3^	0.001*
Group IIb	42 x 10^3^	

## Discussion

Implant restorations and bone act as substantive functional components. Any discrepancies in the implant abutment assemblies may cause adverse effects on both the implant and the bone. The significant mismatch between the implant abutment assembly is microgap [17]. This study found that the pre-machined BioHPP PEEK abutment-Ti implant has lesser vertical microspace and microbioleakage than that of CAD/CAM milled zirconia abutment-Ti implant before and after cyclic loading. Microspace varies in different zones in both groups. In the current study, three regions were considered (front right, center, and front left), and the vertical microspace was found to vary between the two groups.

The size of the microspace varies from 1-50µm, and it is predominantly related to the implant size and torque involved in screwing the abutments [9]. The forces of mastication exerted on the implant prosthesis may cause micromovement at the interfaces. The microspace can increase during the mouth's closing and opening, resulting in pumping at the IA junction. This could create bacterial colonization in the microspace [18].

Rismanchian et al. explained that the type of abutment used also influences the microgap. The mean microspaces of Straumann abutments were 7, 10µm, which was lower than the mean microspace of 2µm for castable abutments. Several factors must be considered when assessing the determinants of passive adaptation prostheses, which include passive fit and methods involved to achieve the same [9].

Hecker et al. demonstrated that the microspace with implant abutment prostheses could vary greatly under certain stress conditions [19]. The smaller gap may exist after repeated stress loading changes, possibly due to wear in the IA junction. Future component loosening or breakage can occur if retightening of prosthetic screws is attempted.

Microspace between the implant abutment interface (IAI) is inevitable. Klotz et al. mentioned that the titanium implant-abutment complex showed a lesser wear pattern than the zirconia because they had similar properties. They also concluded that zirconia abutments tend to have more wear resistance [20]. Following our present study, the mean vertical microspace of CAD/CAM milled zirconia abutments was relatively higher than PEEK abutments. Comparatively, due to the low elastic modulus of PEEK, it has lower wear resistance than zirconia abutments. PEEK also has excellent abrasive resistance [21]. The current study utilized pre-machined Bio-High performance polymer PEEK abutments. This study aims to draw the merits of the newer materials and their usage in the future to decrease complications of microgap and its subsequent failures.

Several studies have also reported that cyclic loading may increase the size of the microspace, especially in the external hex implant system [17]. This study shows that both groups' two internal hex systems showed lesser vertical microgap after cyclic loading than pre-cyclic loading.

In this study, a cyclic stress of 100 N was given to all samples for 150,000 cycles simulating six months of function. After cyclic loading, the BioHPP PEEK abutment showed a lesser vertical microgap than the zirconia abutment. The variations of the microspace were seen in various zones. Independent t-test revealed a statistically insignificant difference between the BioHPP PEEK abutment-Ti implant and CAD/CAM milled zirconia abutment-Ti implant before and after cyclic stress delivery. The vertical microspace between the BioHPP PEEK and titanium implant ranges from 12.9 to 13.76µm before cyclic loading and 9.34 to 10.51µm after cyclic stress delivery.

In contrast, the vertical microgap between CAD/CAM milled zirconia abutment and titanium implant ranges from 17.84 to 18.58µm before cyclic loading and 13.3 to 14.5µm after cyclic loading. The minimal microspace differs between the pre-machined and castable abutments. Baldassarri et al. reported the mean vertical microspace value of 1.6µm, 5.7µm, 8.4µm, and 11.8µm in CAD/CAM milled zirconia abutments on using different implant systems [22].

Cunha et al. reported vertical microspace values of 5.7µm, 9.53µm, and 10.62µm on comparing fit accuracy between CAD/CAM milled zirconia abutments over three implant systems [23]. Compared with the current study, the mean vertical microspace for CAD/CAM milled zirconia abutment titanium implant ranges from 17.8µm to 18.58µm before cyclic loading. In contrast, it ranges from 13.3µm to 14.5µm after cyclic loading.

In certain circumstances, it may be necessary to use a custom-made abutment. It is mainly used for patients with reduced interdental spaces and misaligned implants. Abutments can be processed in various ways, such as casting, milling, and laser sintering. The processing procedure errors may occur due to the customization process and can increase the microspace [24]. The microspaces of zirconia abutments increase when torque values less than the company indicated are applied. The pre-manufactured abutments exhibit smaller microspaces than castable abutments [25]. 

Under static conditions, bacteria can enter the interior of the implant and colonize. Microgaps provide nutrients to the bacteria inside the implant, causing migration from the inner aspect of the implant to the oral environment continuously. Bacteria are, therefore, present around the IAI [25]. In this study, all samples were immersed in SCDB medium, enriched with 1.0ul E. coli, and incubated for seven days at 370 C. As E. coli is a motile, gram-negative anaerobic bacteria, it tends to replicate in an SCDB medium when incubated for 17-18 hours. However, the turbidity of the broth was seen only after three days of incubation at the IAI. This would be mainly due to the minimal microgap, thereby restricting bacterial colonization at IAI.In this study, the microleakage at BioHPP PEEK abutment-Ti implant is considerably lesser than that of CAD/CAM milled zirconia abutment-Ti implant.

Mann-Whitney U test revealed differences in microbial leakage for E. coli among the two abutment types, and the difference was significant. Rismanchian et al.'s study included 4 ITI abutments with different microspaces. They identified the differences in microbioleakage after 5 and 24 hours. They concluded that over time, the effect of microspace on bacterial microleakage gradually decreases until there is no difference [9]. Microgaps allow microleakage of bacteria to persist around the IA and enhance micromotion during function. In addition, micromotion and microleakage cause fretting, deformation, and loss of screws. These complications can increase micro-movements and microgaps, increasing micro-leakage and mechanical damage. Microspace is the root cause of microbioleakage induced by micromotion. Both components influence and promote each other. As a result, bacteria and endotoxins surround IAI, ultimately inducing crestal bone loss of the implant around the neck. Micromovements harm osseointegration due to mechanical injury [25].

The implant abutment transfers occlusal stress from the superstructure to nearby areas through microspace. It causes an undesired rapid loading, resulting in the loosening of the screw [26]. Moreover, Sahin et al. showed that large IAI microspaces lead to high levels of microbioleakage and small removal torque values. The reversal torque must be equal to or greater than the fastening torque. Reducing the removal torque value means the IAI micro-gap promotes screw loosening, leading to microbioleakage [27]. When fixed and functioning in a titanium implant, zirconia abutments produce more microwear than titanium abutments. As a result, zirconia abutments are prone to microchanges in their structure.

Oral bacteria are 1.1-1.5µm in diameter and 2-6µm on average in length. This free entry of bacteria at the IAI results in movements between the implant's interior and the external oral cavity [28]. The microcap space is enough to mobilize the microorganisms and induce colonization in the microspace. Inflammatory activity is initiated and eventually leads to peri-implantitis [29].

The null hypothesis was accepted for vertical microgap before and after cyclic loading and rejected for microbial leakage. Though the null hypothesis for vertical microgap is accepted, the numerical data and P-value revealed variation between Group I and II (before cyclic loading) and Group IA and Group IIB (after cyclic loading). In other words, the vertical microgap is smaller than CAD/CAM milled zirconia abutment during pre- and post-cyclic stress.

The current research had some limitations. Influence of cold welding between implant and abutment was not included. Microspace was measured only on the buccal aspect, and no information about the opposing side was provided. The processing errors could have been avoided, resulting in a good fit of the castable abutments. E. coli was the only microorganism incubated, whereas the oral microbial flora had many microorganisms. Internal hex design was evaluated in both groups. Future studies can consider more internal hex groups to assess microgaps, and microleakage sealant was applied at the microspace, which could reduce microbial existence. Upcoming studies can evaluate the efficacy of new sealants and more literature studies to support the PEEK abutments in clinical practice.

## Conclusions

Under calibrated cyclic stress simulating oral function, BioHPP PEEK could be considered as a short-term provisional implant abutment material due to its reduced E. coli cellular activity and lesser microspace, especially when torqued over titanium alloy implant. This particular variant of PEEK material is very much machinable over titanium alloy implants, which is evident from the reduced implant-abutment interface.
